# Cell-Centric View of Apoptosis and Apoptotic Cell Death-Inducing Antitumoral Strategies

**DOI:** 10.3390/cancers3011042

**Published:** 2011-03-03

**Authors:** Aintzane Apraiz, Maria Dolores Boyano, Aintzane Asumendi

**Affiliations:** Department of Cell Biology and Histology, School of Medicine and Dentistry, University of the Basque Country, 48940, Leioa (Bizkaia), Spain; E-Mail: lola.boyano@ehu.es (M.D.B.)

**Keywords:** apoptosis, classical-chemotherapy, targeted-chemotherapy

## Abstract

Programmed cell death and especially apoptotic cell death, occurs under physiological conditions and is also desirable under pathological circumstances. However, the more we learn about cellular signaling cascades, the less plausible it becomes to find restricted and well-limited signaling pathways. In this context, an extensive description of pathway-connections is necessary in order to point out the main regulatory molecules as well as to select the most appropriate therapeutic targets. On the other hand, irregularities in programmed cell death pathways often lead to tumor development and cancer-related mortality is projected to continue increasing despite the effort to develop more active and selective antitumoral compounds. In fact, tumor cell plasticity represents a major challenge in chemotherapy and improvement on anticancer therapies seems to rely on appropriate drug combinations. An overview of the current status regarding apoptotic pathways as well as available chemotherapeutic compounds provides a new perspective of possible future anticancer strategies.

## 1. Introduction

Cell death tends to occur in a regulated fashion under both physiological and pathological conditions. Apoptosis, historically termed *necrobiosis* or *chromatolysis* [1] and long used as a synonym for *programmed cell death*, is an area of continuously expanding study. The more we know about organelle cross-talk and regulation, the less valid simple classifications become. Pure and watertight cell death pathways are no longer useful. Surface cell-death receptors and mitochondria have been classically considered to be key locations for the induction of apoptosis. Nevertheless, recent studies have revealed new regulated pathways that highlight the importance of other organelles in the modulation of apoptosis.

Interest regarding the apoptotic process, and cell death pathways in general is strongly supported by the urgent need for efficient tumor cell death-inducing agents. According to the World Health Organization (WHO), cancer caused approximately 13% of the total deaths worldwide in 2007, and cancer-related mortality is projected to continue increasing (http://www.who.int/cancer/en/). In general terms, cancer (defined by the WHO as *the uncontrolled growth and spread of cells*) is treated by antiproliferative and/or cell death-inducing drugs. A better characterization of cell death/survival pathways and a more detailed description of the most commonly altered molecules in cancer has allowed the development of molecule-targeted drugs that aim to achieve more efficient anticancer strategies. Imatinib, a tyrosine kinase inhibitor [2], is considered to be the pioneer member of a rapidly expanding field that we will review here.

## 2. Apoptosis: An Historical View

The term *apoptosis* was coined by Kerr *et al.* [3] in 1972, but the concept is much older [4]. In 1860, R. Virchow described a process for “physiological” cell degeneration and tissue degradation distinct from necrosis; he named it *necrobiosis*. Some years later, in 1885, W. Flemming coined the term *chromatolysis* (from Greek *khroma* = color, + *lysis* from lyein = to split) due to the fact that the broken up nucleus ultimately disappeared [1]. Notably, Flemming described this event as a spontaneous cell death and framed it among physiological processes.

By 1972, a continuous loss of cells was assumed to take place in normal tissues in order to balance mitotic cell proliferation. Kerr *et al.* [3] mentioned that, unlike *coagulative necrosis*, the process they described was induced not just by noxious agents, but could also be detected under several physiological and pathological conditions. Moreover, they described a distinct mode of cellular death with ultrastructural features that were consistent with an active, inherently controlled phenomenon [3]. Despite the fact that the studies were based on ultrastructural analysis, Kerr *et al.* included two concepts that would be coined later: (1) apoptosis is an active process, *i.e.*, *ATP-dependent*, and (2) apoptosis has been known as programmed cell death, an *inherently controlled phenomenon*. Some of the typical examples of apoptosis, such as apoptosis-mediated involution of phylogenetic vestiges in the embryo or its implication during normal organ and digit development, are from the 1970s. “Programmed cell death” has been used as a synonym for *apoptosis* to distinguish it from the other primary cell death process: *necrosis*. Recent data support the idea of an organized process behind necrotic cell death [5-7]. Therefore, programmed cell death is no longer proper terminology and the use of terms such as apoptosis, necrosis, or autophagic cell death is encouraged.

## 3. Morphological Characteristics of Apoptotic Cell Death

**Figure 1.**
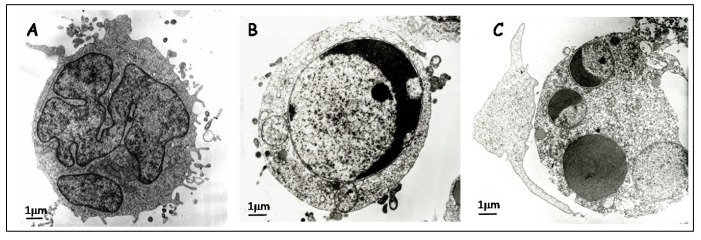
Apoptotic features by electron microscopy. Electronic micrographs of human T-cell acute lymphoblastic leukemia CCRF-CEM cells (5000x). Untreated CCRF-CEM cells (**a**) are characterized by a large nucleus, few cytoplasm and lack of obvious vacuoles. Apoptosis was induced by 4-HPR (3 μM) treatment. Cells undergoing apoptotic cell death (**b-c**) are distinguished by increased vacuolization of the cytoplasm and marginalization of the condensed chromatin (**b**) followed by micronuclei formation (**c**).

Pioneer studies of apoptosis were based on *camera lucida* drawings, and later on electron microscopy and ultrastructural characterization. In 1972, another important concept was named: *apoptotic bodies*. Kerr *et al.* [3] suggested defining *apoptotic bodies* as “small, roughly spherical or ovoid cytoplasmic fragments, some of which contain pyknotic remnants of nuclei”. The apoptotic process was also divided into two discrete stages: (1) apoptotic body formation and (2) apoptotic body phagocytosis and degradation by other cells. General morphological features are currently the same as the ones described in that work. Table 1 contains a detailed list of primary morphological characteristics also illustrated in Figure 1 (for a review see [8]).

## 4. Biochemical Characteristics of Apoptotic Cell Death

Phosphatidylserine exposure in the outer leaflet of the plasma membrane is one, if not the unique, common feature of apoptotic cells, though phosphatidylserine exposure may also be present in processes other than cell death [8]. Phosphotidylserine is a phospholipid that, under general conditions, is “sequestered” in the inner leaflet of the plasma membrane by an ATP-dependent translocase. Elevation of the cytosolic Ca^2+^ concentration, which is common but not specific to the apoptotic process, has been shown to inhibit translocase activity and activate a scramblase (*i.e.*, non-specific, bidirectional lipid flippase), leading to randomized phospholipid distribution in the plasma membrane [9]. Moreover, phosphotidylserine translocation to the outer leaflet has been shown to occur in a cytosolic Ca^2+^-dependent and cytochrome *c* release-, caspase activation-, or DNA fragmentation-independent fashion [10], supporting previous data about apoptosis-independent phosphotidylserine exposure [9].

During the apoptotic process, phosphotidylserine exposure in the outer leaflet has been suggested to activate macrophage-mediated phagocytosis [11], driving an inflammation-free cell clearance (*secondary necrosis* in the case of phagocyte-lacking *in vitro* systems, Table 1). The fact that phosphotidylserine-binding molecules, such as annexin V, can inhibit apoptotic cell clearance supports the role of phosphotidylserine in macrophage activation [9]. Nevertheless, the precise mechanism for apoptotic cell-macrophage/phagocyte interaction remains unclear. T-cell immunoglobulin mucin (TIM) family members, especially TIM-4, were recently proposed as phosphotidylserine-recognizing phagocyte receptors (as commented on by Savill and Gregory [12]), but the study of this process still has a long way to go.

**Figure 2.**
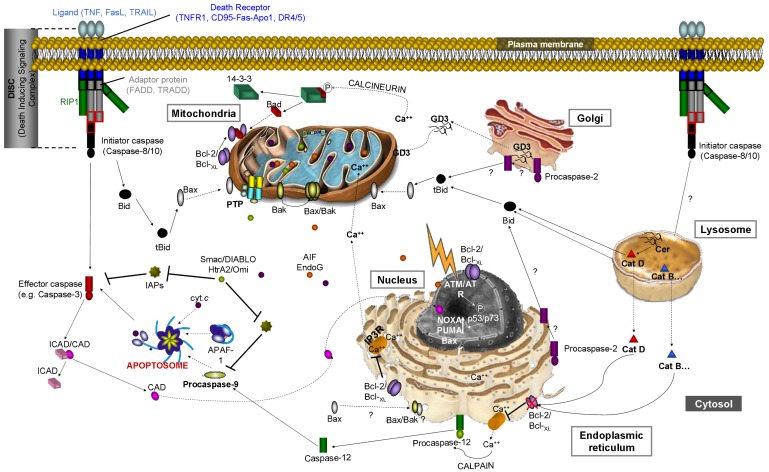
General view of the primary apoptotic pathways in the cell. Death receptor-dependent and –independent signaling pathways as well as signaling connections among different organelles are represented. Inhibitory effects are designed by while activation is designed by solid lined-arrows (→). Dash lines (---) have been used to represent movement and interrogation marks (?) for proposed undetermined steps.

Regardless of common phosphotidylserine externalization and the morphological similarities, several subtypes of apoptosis have been described based on activated biochemical routes. According to the most general scheme, apoptotic processes can be divided into two major groups: extrinsic (or cell surface death-receptor mediated) and intrinsic (or receptor-independent) (Figure 2). The classification of other programmed cell death types, such as perforin/granzyme-mediated pathways (specific for T cells and natural killer cells), is not still clear [13,14].

### 4.1. Cell Death Receptor-mediated Apoptotic Pathway

In the extrinsic pathway, the apoptotic signal is triggered by a ligand binding the corresponding cell surface receptor, followed by formation of the death inducing signaling complex (DISC) (Figure 2). The surface transmembrane receptors belong to the tumor necrosis factor receptor (TNFR) gene superfamily and share similar cysteine-rich extracellular domains and a cytoplasmic domain of about 80 amino acids called the death domain (DD) [15].

Based on current data, there are three well characterized surface transmembrane receptor systems that ultimately form DISC: TNFR1 (activated upon TNF binding), CD95/Fas/Apo1 (activated by Fas ligand (FasL)), and death receptor 4 and 5 (DR4/5; activated by Apo2 ligand (Apo2L), also known as TRAIL or TNF-related apoptosis inducing ligand) [13].

According to the current scenario, death receptors assemble prior to binding their corresponding ligands (TNF, FasL, or TRAIL). Upon receptor-ligand binding, receptors undergo a conformational change that is required for further adaptor protein recruitment (e.g., Fas-associated protein with DD (FADD), TNFR-associated protein with DD (TRADD)). Receptor-adaptor interaction occurs through the DD present in both groups. Adaptor proteins contain not just a DD, but also a death effector domain (DED), which is necessary for the recruitment and activation of cell death effectors, such as initiator caspases (caspase-8 and -10) and the long form of cellular FLICE/caspase-8-like inhibitory protein (cFLIPL). The assembled cell death receptor-ligand-adaptor-effector complex forms the DISC (Figure 2) [13,16]. Among receptor binding proteins, receptor interacting protein 1 (RIP1) is a serine/threonine kinase with a DD domain that seems to be in the crossroads of several pathways and involved in main cellular processes, from proliferation to apoptosis and necrosis. RIP1 has been shown to bind to several death receptors, as well as to DD-containing adaptors like FADD and TRADD, through its own DD domain [17]. According to O'Donnell *et al.* [18], the ubiquitination status of RIP1 could determine its function, switching from a pro-survival molecule in the receptor-bound, polyubiquitinated form to a pro-apoptotic, caspase-8-activating molecule when de-ubiquitinated. Therefore, ligand-mediated death receptor activation does not necessarily result in apoptosis induction.

In regards to the receptor-mediated apoptosis induction scheme, the mechanism for *initiator caspase* activation is still being debated. Mammalian *initiator caspases*, together with *effector caspases* form a family of 11 cysteine-dependent aspartate-directed proteases synthesized as inactive zymogens. Activation of the zymogens requires the removal of a prodomain present in all caspases as well as the cleavage at the intersubunit linkers into the large (p20) and small (p10) subunits (for a comprenhensive review of caspases in apoptosis as well as other processes, read [19]). Caspases are currently considered to be the prime mediators of the apoptotic program execution although the relevance of other proteases is also increasing [20].

Back to the mechanisms involved in initiator caspase activation, Guicciardi and Gores [16] described the main hypothesis, referred to as “the induced-proximity model”, “proximity-induced dimerization model” (no cleavage required), and “conformational change-mediated activation model”. Regardless of the mechanism of activation, once activated the enzyme is released into the cytosol in an active heterotetrameric form that triggers a proteolytic cascade. Two downstream pathways are the most likely: (1) direct activation of *effector caspases* (e.g., caspase-3, -6, and -7), or (2) cleavage of the pro-apoptotic BH3-only protein Bid, which would further activate the mitochondrial apoptotic pathway (Figure 2). Current data suggest that downstream pathway-selection may be linked to the amount of active initiator caspase (e.g., active caspase-8) [16].

### 4.2. Cell Death Receptor-independent Apoptotic Pathways

Cell death receptor-independent apoptotic pathways, also known as intrinsic apoptosis pathways, are initiated by a diverse set of non-receptor-mediated stimuli that act directly on targets within the cell. Signals that drive the intrinsic apoptosis pathway can be triggered by the absence of certain growth factors, hormones, or cytokines; the presence of negative environmental conditions (e.g., hyperthermia, hypoxia) or damaging agents, such as radiation, free radicals, and drugs; or viral infections [14].

The intrinsic apoptotic pathway has been linked to mitochondrial damage and termed the *mitochondrial apoptotic pathway* [21,22]. However, more and more evidence indicates the involvement of other organelles, including the endoplasmic reticulum (ER), nucleus, or lysosomes as *intrinsic apoptotic pathway checkpoints* [13,23,24].

#### 4.2.1. Mitochondria-triggered Intrinsic Apoptosis Pathway

Mitochondria are the main “energy factories” in aerobic systems and, therefore, essential for cellular survival. Mitochondrial membrane potential (Δψ_m_) dissipation and mitochondrial membrane permeabilization (MMP) are considered two of the main events in the so-called “*point-of-no-return*” for cellular, but not necessarily apoptotic, death [8].

Mitochondria are complex organelles with two main and unique characteristics: (1) the presence of its own genetic material (circular DNA) that encodes several, although not all, proteins of the mitochondrial respiratory chain and (2) a double membrane structure. The double membrane structure is essential for proton (H^+^) gradient formation, which ultimately drives energy generation (adenosine-triphosphate, ATP, molecules). The proton (H^+^) gradient, and thus Δψ_m_, originates from the accumulation of H^+^ in the intermembrane space (IMS) via active pumping from the mitochondrial matrix (see Figure 6).

During intrinsic apoptosis, direct or indirect (e.g., as secondary damage after Ca^2+^ release from the ER) mitochondrial damage is characterized by a loss of Δψ_m_. Depletion of the H^+^ gradient has been classically described as the consequence of two events: (1) impaired oxidative phosphorylation (e.g., by direct inhibition of electron transport chain complexes) and (2) formation/opening of any of the pores in the mitochondrial membrane system, such as the permeability transition pore (PTP), or Bax/Bak pores. Moreover, mitochondrial outer membrane (OM) permeability also drives the release of several pro-apoptotic proteins, including cytochrome *c*, Smac/DIABLO, HtrA2/Omi, apoptosis inducing factor (AIF), and endonuclease G [14,25].

**Figure 3.**
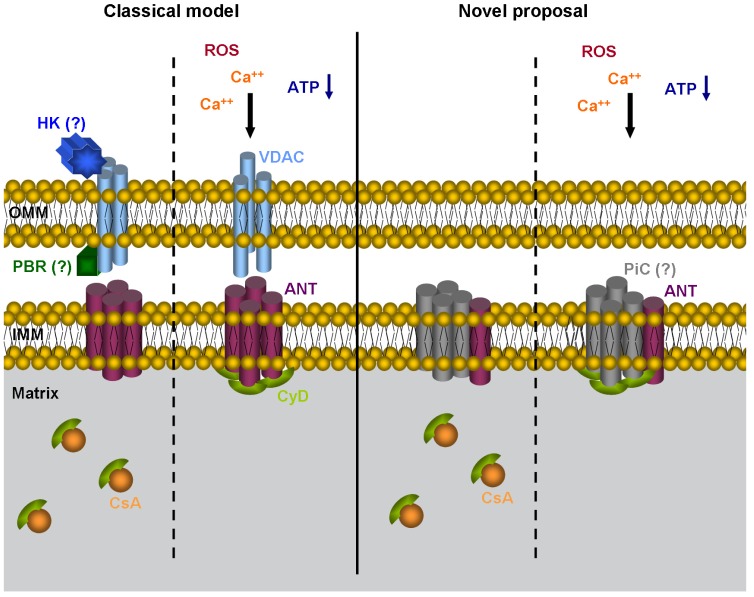
Comparison of the classical (left side) and novel models (right side) of the mitochondrial permeability transition pore (PTP). The role of hexokinase (HK), peripheral-type benzoazepine receptor (PBR) and mitochondrial phosphate carrier (PiC) in the mitochondrial transition pore formation/function are still controversial [27,31].

According to the classically accepted, though controversial, model, mitochondrial permeability transition (MPT) is based on opening of the PTP protein complex. Classical (vs. newly proposed) PTP (Figure 3) is a protein complex formed by the voltage-dependent anion channel (VDAC) located in the mitochondrial OM, the adenine nucleotide translocator (ANT) in the mitochondrial IM, cyclophilin D (CypD) in the mitochondrial matrix, and several other proteins at contact sites between the mitochondrial outer and inner membranes [26,27]. Despite the fact that most of the data are from *in vitro* studies, cytosolic Ca^2+^, low ATP, and reactive oxygen species (ROS) are the most accepted inducers of PTP opening, whereas cyclosporin A reverts the opening in most cases by sequestering CyD in the mitochondrial matrix [27]. Current data, however, indicate that VDAC is dispensable for MPT-mediated cell death [28]; on the other hand, CyD is gaining importance in mitochondria-mediated cell death and especially, in ischaemia/reperfusion-mediated cardiac injury [29,30]. Nevertheless, recent studies indicate that CyD is not a common mediator in mitochondrial-mediated apoptosis and may be primarily involved in ROS or Ca^2+^ overload-induced necrotic cell death [30].

Consequently, a second PTP-model has been proposed (Figure 3) in which VDAC is not a part of the PTP complex and mitochondrial phosphate carrier (PiC) is incorporated [31]. Therefore, not just the composition of the PTP but also its role in cell death processes is now under discussion. In general, PTP opening is known to result in the diffusion of low-molecular weight solutes (up to 1.5 kDa) across the inner membrane (IM), resulting in mitochondrial swelling and a loss of Δψ_m_ In this scenario, rupture of the OM would be a consequence of IM swelling [27,31]. Interestingly, Bcl-2 family members seem to control MPT; Bcl-2/Bcl-_XL_ has been shown to inhibit VDAC and ANT activity in liposome experiments [27], but the relevance of this data is difficult to evaluate in the absence of a clear working model.

Bak and Bax belong to the Bcl-2 protein family. This family includes a large number of pro- and anti-apoptotic proteins that share one or more Bcl-2 homology (BH) domains, which is important for heterodimeric interactions among members of the Bcl-2 family [13,32]. The founding member of the family, the proto-oncogene Bcl-2, was first identified in the 1980s [33,34]. Bcl-2 expression was shown to block cell death, unlike other proto-oncogenes known at that time that typically promoted cellular proliferation [35]. The first pro-apoptotic homolog, Bax, was identified some years later by its interaction with Bcl-2 [36]. The Bcl-2 family currently comprises roughly 20 members that are classically divided into three main groups based on their pro- or anti-apoptotic function and the presence of BH domains: Group I consists of anti-apoptotic Bcl-2 members (e.g., Bcl-2, Bc1-_XL_, Mcl-1), Group II contains BH-1, BH-2 and BH-3 presenting pro-apoptotic members (e.g., Bax, Bak) and Group III comprises BH-3 only members (all of them with pro-apoptotic function, such as Bad or Bid) [37].

According to the generalized *rheostat-model* [38], upon stimulation, the ratio of anti-and pro-apoptotic Bcl-2 family members (e.g., Bcl-2/Bax) sets the threshold of susceptibility to apoptosis. Briefly, the pro-apoptotic Bak seems to be inserted into the OM even when inactive [39], whereas Bax monomers can be found in the cytosol or loosely associated with the OM when not activated. Bax translocation to the mitochondrial OM takes place during the activation process [32,40]. At the mitochondrial level, the interaction of anti-apoptotic (e.g., Bcl-2) and pro-apoptotic members (e.g., Bax) appears to inhibit Bax-Bak-mediated pore formation at the mitochondrial OM, preventing Δψ_m_ loss and the release of pro-apoptotic proteins (e.g., cytochrome *c*, Smac) into the cytosol (Figure 2). (For a review see [32]).

The release of mitochondrial IMS proteins (*i.e*., cytochrome *c*, Smac/DIABLO, HtrA2/Omi, AIF, and endonuclease G) leads to activation of the execution phase. Once in the cytosol and in the presence of dATP/ATP, cytochrome *c* binds and activates apoptotic protease-activating factor 1 (Apaf-1) and procaspase-9, forming the *apoptosome*. Apoptosome formation is required for procaspase-9 activation and caspase-3/caspase-7 activation (for a review see [41]). On the other hand, Smac/DIABLO and HtrA2/Omi have been reported to inhibit cytosolic inhibitor of apoptosis proteins (IAPs) [14], which block caspase-9, -3, and -7 activation [42]. The remaining IMS proteins (*i.e.*, AIF and endonuclease G) are in charge of DNA fragmentation and/or chromatin condensation in the nucleus, forming the typical apoptotic “DNA ladder” structure. Among the endonucleases, cytosolic caspase-activated DNase (CAD) is involved in the caspase-dependent cell death mechanism, whereas AIF and endonuclease G induce caspase-independent cell death [14].

Despite being a canonical intrinsic apoptotic pathway, mitochondria are far from the only elicitor organelles. As summarized in Figure 2, ER stress can also trigger intrinsic apoptosis through a pathway generally, though not compulsorily, involving secondary mitochondrial damage. Furthermore, DNA damage may also lead to the activation of intrinsic apoptosis as briefly described in a following section.

#### 4.2.2. Endoplasmic Reticulum-triggered Intrinsic Apoptotic Pathway

Anti-apoptotic Bcl-2 members, such as Bcl-2 itself or Bcl-_XL_, can be found not just at the mitochondrial OM, but also at the ER (Figure 2). Pro-apoptotic Bax/Bak has also been found at the ER, and recent work supports the importance of the ER and ER-localized Bcl-2 family proteins as intrinsic apoptotic pathway checkpoints [13,22,24]. The ER is the main organelle for Ca^2+^ storage and protein synthesis and the first stop for proper polypeptide folding. When the capacity of the ER to fold proteins properly is compromised or overwhelmed (also known as ER stress), the unfolded protein response (UPR) is activated. Sustained or strong ER stress leads to metabolic arrest, which is followed by autophagy or the induction of apoptosis [22,24,43]. Ca^2+^ release from the ER has long been known to play a secondary messenger role in the induction of mitochondrial damage. Recent studies indicate Bcl-2 family members as guards that control apoptosis originating with the ER (through Ca^2+^ efflux regulation), as well as autophagy [22]. However, Bcl-2 family proteins do not merely act at the mitochondrial level.

#### 4.2.3. Nucleus (DNA Damage) Triggered Intrinsic Apoptotic Pathway

The capacity of DNA damage to induce apoptotic cell death is based on three main observations [44]:

- The hypersensitive apoptotic responses of DNA repair-deficient cells (e.g., cell lines defective in O6-methylguanine-DNA methyltransferase (MGMT))
- The ability of modified nucleotide precursors incorporated into DNA to induce apoptosis
- Electroporation of restriction enzymes into cells induces double strand breaks (DSBs) in DNA that trigger apoptosis but not necrosis

Despite being the better-known type of DNA damage, DNA DSBs are far from the only type of DNA damage. In fact, DNA single strand breaks, O^6^-methylguanine formation, base *N*-alkylations, bulky DNA adducts, DNA cross-linking, and lesions caused by O^6^-chloroethylating agents (such as the anticancer drug *cisplatin*) may or may not cause DSBs but induce programmed cell death [44].

Thus, nuclear DNA damage is another example of an intrinsic apoptotic pathway, in which the DNA repair system and protein-modifying enzyme poly(ADP-ribose) protein (PARP)-1 play a critical role [45]. PARP-1, also known as poly(ADP-ribose) synthetase and poly(ADP-ribose) transferase, is an abundant nuclear protein involved in the DNA base excision repair (BER) system. In the presence of mild DNA damage, PARP-1 has been shown to bind DNA breaks and transfer 50–200 residues of poly(ADP-ribose) to itself and acceptor proteins [46]. In contrast, excessive DNA damage generates large branched-chain poly(ADP-ribose) polymers, leading to the activation of a cell death program not yet fully characterized (and applied mainly to cells in the nervous system). According to the most common hypothesis, PARP-1-mediated cell death involves energy depletion [47] and AIF translocation from the mitochondria. However, the precise mechanism of PARP-1-dependent AIF translocation remains unclear [45].

A better characterized and more generalized pathway is the one following DSBs and triggered by ataxia telangiectasia mutated (ATM) and ataxia telangiectasia and Rad3-related (ATR) protein activation [48]. Downstream of ATM activation, other proteins (*i.e.*, CHK1, CHK2, MDM2, H2AX, or the well-known tumor suppressor p53) amplify the signal, leading not only to cell death, but also cell cycle arrest. Briefly, ATM-dependent p53 phosphorylation stabilizes p53; further cell cycle arrest by downstream up-regulation of the cyclin-dependent kinase inhibitor p21 or the induction of apoptosis, which occurs via the activation of several pro-apoptotic proteins (e.g., Bax or p53 up-regulated modulator of apoptosis (PUMA)), seems to be dependent on the p53 activation level. In the absence of active p53, which is a common mutation among cancer cells, CHK1 and CHK2 have been described as inducing apoptosis (not cell cycle arrest) through a pathway involving p73 activation [44].

#### 4.2.4. Mitochondria, ER, Nucleus: Why Not Another Organelle?

Cells are well-organized and compartmentalized entities in which different organelles play distinct and indispensable roles. Nevertheless, discoveries during the last few years support the importance of communication and interaction between organelles for appropriate cell function and fates. Regarding apoptotic cell death, organelle and pathway interactions are summarized in Figure 2. Plasma membrane receptors, mitochondria, the ER, and nucleus have been shown to interact in the complex cellular death process. Information is still lacking concerning the possible role of Golgi or lysosomes in the activation of intrinsic apoptosis.

From a simplistic point of view, the Golgi apparatus is the glycosylation center and vesicular trafficking coordinator in cells. In fact, the strongest evidence of the Golgi apparatus as a point of apoptosis induction is the induction of apoptosis by α-mannosidase II inhibition [23]. Despite the lack of well known Golgi-specific stressors, local caspase-2 [49] and GD3 synthase activation, which is involved in ganglioside synthesis [50], are Golgi-damage responses that have been characterized. GD3 is a complex glycolipid belonging to the sphingolipid family, generally described as sphingoid-based lipids (for a review see [51]). As pointed out by Hicks and Machamer [52], an interesting possibility in Golgi-mediated stress signaling could be the mediation of lipid scrambling in the Golgi membrane bilayers, similar to phosphatidylserine exposure at the plasma membrane during apoptosis. GD3, which is normally synthesized on the luminal face of the Golgi membranes, translocates to the mitochondria during apoptosis, where it is thought to induce MMP [53,54]. Interestingly, if active GD3 synthase is retained in the ER, GD3 is not transported to the mitochondria and the apoptotic stimulus is abrogated [55]. Another role of the Golgi in apoptotic cell death is linked to the presence of TNFR-1 [56] and Fas receptor/APO-1/CD95 [57] in this organelle. Nevertheless, whether the death receptors translocate to the plasma membrane upon apoptotic stimulus, or whether the plasma membrane-initiated death signal is transduced to the Golgi along the endocytic route, is unclear [58].

Lysosomes, cellular “garbage bins” or “suicide bags”, may also be involved in apoptotic cell death. Lysosomes contain large amounts of catabolic hydrolases with optimum acidic pH, and their roles in autophagy, apoptotic cell clearance, and cell death have been extensively studied over the last few years [23,59]. Massive lysosomal membrane rupture has been found to lead to necrotic cell death, whereas moderate lysosomal membrane damage has been shown to induce apoptotic cell death [60,61]. In fact, lysosomal membrane permeabilization (LMP) is now considered a parallel phenomenon to MMP. Moreover, several lysosomotropic detergents (e.g., MSDH, siramesine), oxidative stress, sphingosine (backbone of the sphingolipid structure), and anticancer drugs (e.g., paclitaxel, etoposide, resveratrol), among others, have been reported to induce LMP [59]. As an example of the role of the lysosome in apoptotic cell death, hereditary deficiencies in lysosomal enzymes, such as Farber's disease (caused by ceramidase deficiency, resulting in ceramide accumulation), are characterized by increased apoptosis [62].

Cathepsins (lysosomal proteolytic enzymes that include cysteine, serine, and aspartate proteases) have been extensively implicated in lysosome-mediated apoptotic cell death [59,63,64]. Proteases in this group are synthesized as inactive precursors, glycosylated post-translationally, and directed towards the lysosomal compartment by the mannose-6-phosphate receptors. Acid sphingomyelinase activity-mediated ceramide, one of the better-known cell death-inducing sphingolipids, has been described as the main cathepsin D activator in the lysosomal lumen [65], but characterization of the cathepsin-activating molecule has not advanced. Several studies point out an important role for cathepsins, especially cathepsin B, in TNF-mediated [66,67] and TRAIL-mediated [68] apoptosis. Cathepsin B has been suggested to be a substrate for caspase-8 in this pathway [66]. However, these results must be interpreted with caution due to experimental difficulties with the assays carried out in *cell-free* systems [64,69]. Furthermore, Klaric *et al.* [70] recently published a study in which cathepsins were not essential for TNF-induced cell death.

In order to achieve their role as cell death effectors, activated cathepsins must be released into the cytosol where the pro-apoptotic cascade can be propagated by caspase-dependent or -independent pathways [59]. Again, there is not much information regarding cathepsin substrates in the cytosol. Bid, a pro-apoptotic BH3-only member of the Bcl-2 family, is the most characterized cathepsin substrate [63,64,69]. As already mentioned in Section 3.1, Bid has long been known as a caspase-8/10 substrate that links death receptor-mediated cell death to mitochondrial damage (Figure 2). Cathepsin-mediated (especially cathepsin B, but also L, S, and K) Bid cleavage (tBid) and activation [71,72] represents a second role for Bid in the lysosomal to mitochondrial damage connection (Figure 2). *Recently*, Bid-deficient animals have been shown to undergo cathepsin-dependent cell death [73]. This finding suggests the presence of cathepsin substrates other than Bid. In agreement with this, Droga-Mazovec *et al.* [74] found that cathepsins also degrade the anti-apoptotic Bcl-2 proteins Bcl-2, Bcl-_XL_, Mcl-1, and XIAP.

The role of caspases in the lysosomal-apoptotic pathways is controversial. Though several studies suggest caspase-8, and to a minor extent caspase-2, -3, or -9, as cathepsin substrates (summarized in [63]), other studies mention a possible experimental mistake as the source of false positive substrate detection [64,69]. However, several studies support caspase activation downstream from cathepsin release into the cytosol (for review see [59]).

### 4.3. Perforin/Granzyme-mediated Apoptotic Pathway

Special mention is required for the perforin/granzyme apoptotic pathway, a mechanism detected in cytotoxic T lymphocytes (CTL) and natural killer (NK) cells, which induce apoptotic cell death in neighbor “dangerous” (*i.e.*, virus-infected or transformed) cells [14,75,76].

**Figure 4.**
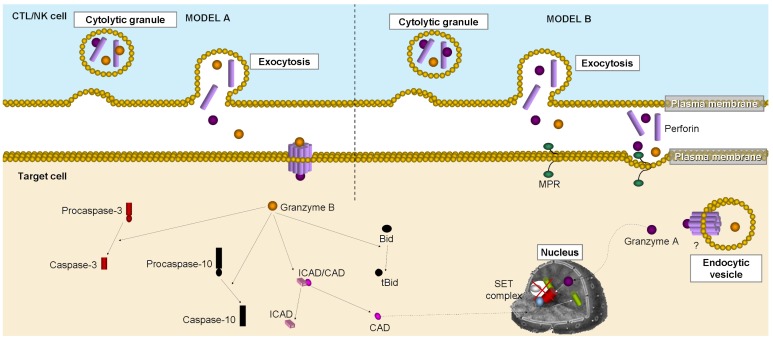
Perforin/granzyme-mediated apoptotic cell death. Despite specific cytotoxic T lymphocytes (CTL) and natural killers (NK), this type of apoptosis is essential for the defensive role of our immune system. Model A and Model B represent proposed strategies for granzyme introduction in the target cell. Perforin is required for the granzyme-mediated apoptotic death of the target cell but it is no clear whether it directly forms pores in the plasma membrane of the target cell (Model A) or whether perforin/granzyme internalization occurs via receptor-mediated endocytosis (Model B). Mannosa-6-phosphate receptor has been recently proposed as a candidate for this process (reviewed in [14,75,76]).

CTL and NK cells are important members of the defense mechanism, a role that is achieved not just through the perforin/granzyme pathway, but also through a second mechanism involving the plasma membrane death receptor (e.g., Fas/Apo-1/CD95) pathway. Regarding the perforin/granzyme pathway, the mechanism engages a transmembrane pore-forming molecule (perforin) and the release of cytoplasmic granules containing serine proteases (granzymes) capable of cleaving and activating proteins (e.g., procaspase-10, inhibitor of CAD (ICAD)) on the target cell. Since the discovery of perforin in the early 1980s, several models have been proposed (summarized in [75]) for this mechanism, but it has yet to be fully characterized. According to current knowledge, granzymes A and B are the most important components within the granules [77] and both the caspase-dependent and -independent pathways can be activated at the target cells [14,76] (Figure 4).

Briefly, granzyme B activates proteins, such as procaspase-10 or ICAD [78,79], probably by cleaving them at aspartate residues [80]. Other reports indicate granzyme B-dependent cleavage of Bid [81], or even direct executer caspase-3 activation [82], as a mechanism of apoptotic death induction. On the other hand, granzymes A and M seem to activate the caspase-independent death pathway via direct DNA damage. In this case, granzyme A cleaves the SET complex, driving the release of NM23-H1, a tumor suppressor DNase [83].

Though perforin is known to be required for granzyme-mediated apoptosis, the precise mechanism for granzyme uptake remains controversial. Perforin pore-mediated direct granzyme uptake and perforin/granzyme-including endosomal vesicle incorporation are just some of the proposed mechanisms (Figure 4) [76].

## 5. Apoptosis-inducing Agents in Cancer Therapy

The apoptotic death of tumor cells is always the desirable effect of therapeutic agents. Optimal treatment should be based on the specific alteration of the target cell compared to non-malignant cells. Nevertheless, this optimal scenario is rare in cancer; malignant progression of the cell may be caused by thousands of different modifications. In addition, tumor cells often have high proliferation rates that, together with their acquired immortality, provide a favorable atmosphere for mutations. Therefore, major efforts are focused on the detection of frequently altered pro-survival signaling pathways, whereas most classical chemotherapeutic agents and radiation pursue DNA damage and/or cell proliferation inhibition-mediated control of the disease (for a review see [84]).

On the other hand, bioavailability (*i.e*., concentration of the active drug surrounding target tumor cells in the body) is often a limiting factor in chemotherapy, despite new nanoparticle-mediated drug delivery systems [85]. Achieving the desirable drug concentration is an essential aspect of chemotherapy as many drugs may induce different biological processes (e.g., cell cycle arrest, autophagy, or necrosis instead of apoptotic cell death [86,87]) in a dose-dependent manner.

According to the 13th Anatomical Therapeutic Chemical (ATC) classification (corresponding to 2010), antitumoral agents (L01 subclass) are divided into five different groups: (1) alkylating agents, (2) antimetabolites, (3) plant alkaloids and other natural products, (4) cytotoxic antibiotics and related substances, and (5) other antineoplastic agents (Table 2).

ATC classification is based on the primary therapeutic use of the main active ingredient. In regard to chemotherapy design, two approaches are currently used: (1) classical drug synthesis based on the empirical chemopreventive/antineoplastic effect of certain compounds, often with natural origin (e.g., retinoic acid, a natural derivative of vitamin A) and (2) cell death-inducing agents designed with a focus on specific molecular targets with relevance on tumor cell survival (e.g., inhibitors of anti-apoptotic Bcl-2 family members). Chu and DeVita [88] provided an extensive physician-oriented review of currently used chemotherapeutic drugs, including the mechanism of action, indications, drug manufacturers, and marketed names.

### 5.1. Classical Chemotherapy: Against the Malignant Phenotype

This group includes a long list of compounds that affect any of the tumorigenic characteristics of the cell (e.g., uncontrolled proliferation, immortality, acquired migration capacity, invasion) without a precise (or known) molecular target. We will focus on chemotherapeutic agents that mainly lead the tumor cell to an apoptotic death (Table 3).

#### 5.1.1. DNA-Damaging Agents

**Figure 5.**
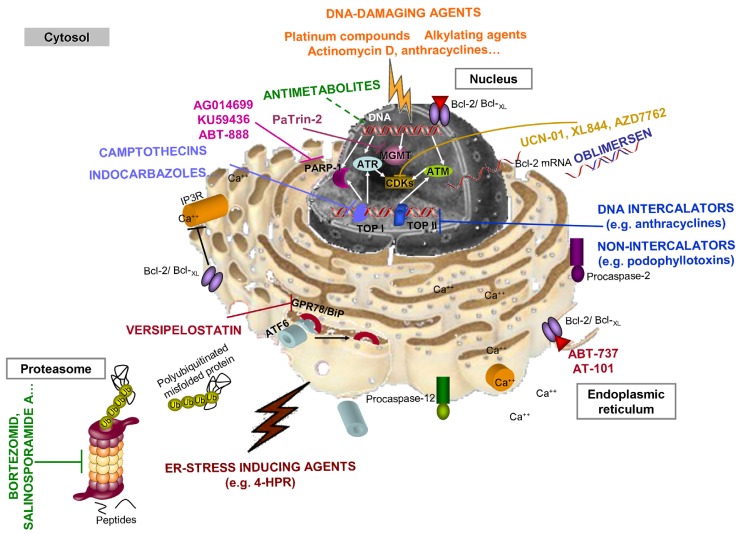
Drugs targeting DNA, endoplasmic reticulum, or proteasome. The figure represents an overview of compounds aimed to induce tumor cell death by interacting with specific molecules (as shown in the figure) or by inducing DNA damage.

DNA-damaging agents are double-edged swords; they have a well-known carcinogenic capacity but are used as antitumoral drugs. Despite this negative aspect, this group is the largest among antineoplastic compounds (Figure 5). Far from simple, the term “DNA damage” includes a broad range of damage already mentioned in Section 3.2.3.

Metal-based drugs, especially platinum compounds (L01XA group according to the current ATC classification), are perhaps the most characteristic DNA-damaging therapeutic drugs with cisplatin (cis-diamminedichloroplatinum (II)) as the pioneer member of the group. Metal-based drugs include not just platinum compounds, but also recently developed gold, ruthenium, and copper-based agents (for a general overview of the field, see [89]). Briefly, the antitumoral activity of platinum compounds is thought to rely on their ability to form different types of adducts with DNA, which blocks replication and transcription and ultimately drives cell death. Nevertheless, platinum compounds may also act through other mechanisms. Cisplatin, for example, has been shown to modulate several proteins by interacting with their thiol groups. Moreover, cellular detoxification of cisplatin seems to be mediated by its reaction with sulfur groups [90], although recent data argue against the generally accepted direct interaction between glutathione and cisplatin molecules as the principal mechanism [91]. Regarding cellular detoxification, intracellular glutathione levels have been linked to resistance against this compound [90]. Observed cisplatin-mediated oxidative stress may also be a consequence of its capacity to “sequester” reduced glutathione molecules [90,92]. (For a comprehensive review regarding cisplatin and other platinum compounds (*i.e.*, carboplatin, oxaliplatin, Lipoplatin, Lipoxal, and nedaplatin), their current use in chemotherapy, adverse effects, or ongoing clinical trials see [93,94]).

Alkylating agents (L01A group according to the ATC classification) include a long list of compounds with the capability to generate DNA base damage [84] via direct interactions that lead to DNA crosslinking, DNA strand breaks, and abnormal DNA base pairing. Similar to platinum compounds, a lack of discrimination between normal and tumor cells leads to important side effects upon treatment with alkylating agents. (For a complete review regarding better known alkylating agents (*i.e.*, nitrogen mustards, ethylene imines, alkyl sulfonates, and nitrosoureas) see [95].

Cytotoxic antibiotics, such as actinomycin D, doxorubicin, and mitomycin C, are natural or natural-derivative compounds with several applications, including chemotherapy. These compounds damage DNA or DNA-related processes via several mechanisms, such as binding specific DNA sequences (followed by inhibition of RNA polymerase-mediated mRNA synthesis), inhibiting topoisomerase activity (mentioned in Section 4.2.2.), or inducing DSBs [96,97]. According to the ATC classification, antitumoral antibiotics are divided into three different groups (Table 2): (1) actinomycins, (2) anthracyclines and derivatives, and (3) others.

Actinomycin D, the most significant actinomycin, is a natural antibiotic produced by the bacteria *Streptomyces antibioticus*. Actinomycin D binds GC-rich DNA duplex sequences, non-duplex single-stranded structures, and hairpin structures, consequently inhibiting RNA polymerase activity. The antibiotic was also recently shown to bind the cMyc oncogene promoter G-quadruplex structure, which would represent a novel mechanism for its antitumoral activity [97].

Anthracyclines, such as daunorubicin and doxorubicin (doxorubicin hydrochloride or Adriamicin), are naturally synthesized by different strains of the bacteria *Streptomyces peucetius* or semisynthetic (e.g., idarubicin and epirubicin) (for a comprehensive review see [96]). The mechanism of action involves, but is not limited to, (1) intercalation into DNA, (2) free radical generation, 3) DNA binding and alkylation, 4) DNA cross-linking, and 5) topoisomerase II inhibition [96].

Mitomycins are a group of antibiotics isolated from *Streptomyces caespitosus* or *Streptomyces lavendulae* and present a high capacity for crosslinking DNA to 5′-CpG-3′ sequences [98]. Among mitomycins, mitomycin C exerts the highest activity against tumor cells and is commonly used in cancer therapy [99]. Reduction of this compound is necessary for its activation, a process that may drive superoxide production and, ultimately, oxidative stress. In fact, a deficiency of activating enzymes, such as NAD(P)H oxidoreductase, has been linked to the development of clinical resistance against mitomycin C [98]. Bleomycins are also synthesized by several *Streptomyces* species, and their antitumoral activity relies on sequence-selective, metal and oxygen-dependent oxidative cleavage of DNA and RNA molecules with subsequent induction of oxidative stress (for a review see [98]).

#### 5.1.2. Oxidative Stress-mediated Apoptotic Cell Death

Imbalance among reactive oxygen/nitrogen species (ROS/RNS) production and neutralization creates an oxidative environment in the cell that may ultimately damage lipids, proteins, and DNA/RNA molecules. An oxidative environment and cancer have been often associated [100] based on the following observations: (1) ROS and oxidative stress may induce cancer; (2) transformed cells appear to generate more ROS than normal cells; (3) cellular antioxidant systems (e.g., thioredoxin system) are often amplified in malignant cells; (4) the stimulation of cellular proliferation (e.g., by growth factors) often involves increased ROS production, and (5) partial selectivity of diverse antitumoral agents against malignant cells is based on the intrinsic augmented oxidative stress of tumor cells, as they act by pushing tumor cells beyond their capacity to handle such stress. Therefore, the relationship between oxidative stress and cancer is at least complex, and at some points controversial.

A large number of antineoplastic treatments/compounds, such as cisplatin, mitomycin C, and radiation, induce an increase of the cellular ROS/RNS content, and this secondary effect may be involved in their anticancer activity, but it also represents a limiting factor in their therapeutic application by inducing important side effects. Free radical scavengers and antioxidants have been shown to decrease undesirable toxic effects [101,102], though they may also decrease the activity of the antitumoral drug, presumably whenever oxidative stress induction is an important mechanism of action [103]. Wang and Yi [104] reviewed the arguments in favor and against the use of pro-oxidant compounds in cancer therapy.

**Figure 6.**
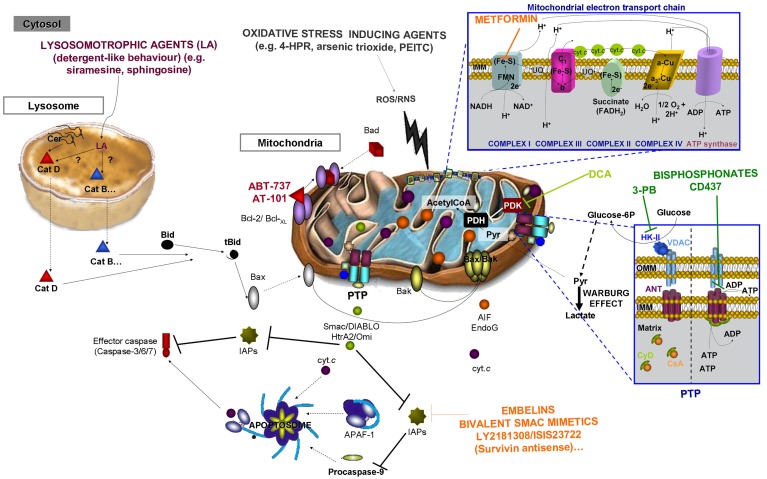
Drugs targeting mitochondrion or lysosome. The figure represents an overview of compounds aimed to induce tumor cell death by interacting with specific molecules (as shown in the figure), by inducing oxidative damage or permeabilization of the lysosomal membrane.

Despite some controversial aspects, increased cellular ROS/RNS content (or decreases in the cellular antioxidant system) seems to be important for the induction of malignant cell death by several antitumoral drugs/treatments, including 4-HPR [105], 2-deoxy-D-glucose [106], NSC-741909 [107], arsenic trioxide, imexon, photodynamic therapy, β-phenylethyl isothiocyanate (PEITC), and disulfiram [104]. Notably, many of these compounds still lack a precise mechanism or target molecule, though mitochondrial damage persists as the most common and evident effect (Figure 6; for a comprehensive review regarding the therapeutic potential of mitochondrial-damaging oxidative stress inducers see [108]).

### 5.2. Specific Molecule-targeted Agents

Understanding the molecular etiology of the disease and the intrinsic regulation of pro-survival pathways in the cell provides necessary tools for chemotherapeutic drug development. In this regard, imatinib (also known as CGP-57148(B) or STI-571, and marketed as Gleevec or Glivec) represents the first member of the specifically targeted drugs by inhibiting the tyrosine kinase enzyme ABL [109]. This and other examples will be classified according to their target proteins or enzymatic activities, with special emphasis on those with inherent pro-apoptotic activity (Tables 2 and 3).

#### 5.2.1. Strategies Against Bcl-2 and Related Anti-apoptotic Family Members

Bcl-2 and related family members have been a potential therapeutic target since first being implied in tumor cell survival [35]. The previously mentioned “rheostat model” [38] supports the importance of down-regulating anti-apoptotic Bcl-2 family members (*i.e.*, Bcl-2, Bcl-XL, Bcl-W, Mcl-1, Bcl-2A1, and Bcl-B) to promote apoptotic tumor cell death.

Gossypol, a polyphenolic compound found in the cotton plant, was the first inhibitor of antiapoptotic Bcl-2 family members, such as Bcl-XL [110], to be described, though its antineoplastic effects have been known since the 1960s [111]. Being a naturally racemic mixture of (+) and (−) enantiomers, the levo (-) form has shown sustained growth-inhibitory effects in the presence and absence of serum (*vs*. the (+) form, which has decreased activity in the presence of serum [112]). In fact, (-)-gossypol enantiomer separation leads to R-(-)-gossypol-acetic acid (AT-101) development (Figure 6), which has shown high affinity to Bcl-2 and Mcl-1 [113].

ABT-737 (A-779024), which was found in nuclear magnetic resonance (NMR)-based screening, is another example of Bcl-2, Bcl-XL, and Bcl-w-inhibiting molecules that do not initiate apoptosis but enhance the pro-apoptotic effects of other compounds [114]. Bioavailability problems have supported the discovery of another Bad-like BH3 mimetic compound, ABT-263, which is orally available, unlike ABT-737 [115]. These and other BH3 mimetic compounds act by sequestering Bcl-2 (or other antiapoptotic members) molecules (Figure 6) and, therefore, promote the activity of the pro-apoptotic members of the family (e.g., Bax, Bak, Bim).

Molecular cloning of the Bcl-2 sequence [116,34] allowed the development of a unique type of Bcl-2 antisense oligonucleotide drug by the early 1990s [117] that is currently known as oblimersen sodium or G3139 (marketed as Genasense). This compound inhibits Bcl-2 protein synthesis by inactivating Bcl-2 mRNA (Figure 5) and has shown chemosensitizing effects in combination with other conventional chemotherapeutic drugs, especially in chronic lymphocytic leukemia patients [118] (for a review see [113]).

#### 5.2.2. DNA-Damaging Agents

Some DNA-damaging agents, known as *antimetabolites*, present clear target molecules and, therefore, must be included in this section. Antimetabolites can be defined as “substances that closely resemble metabolites and therefore interfere with physiological reactions involving them” [119]. Antimetabolites applied in chemotherapy (Tables 2 and 3) act by interfering with DNA synthesis. Briefly, folic acid analogues or antagonists, such as methotrexate or pemetrexed, inhibit dihydrofolate reductase [119,120], depleting cells of reduced folates, which are required for purine synthesis. Other antimetabolites are purine or pyrimidine analogues, “fraudulent” nucleotides that are incorporated into DNA during DNA synthesis and lead to defective DNA replication (Figure 5). In this regard, antimetabolites may not only induce apoptotic cell death, but also slow down the proliferation rate or cell cycle arrest (for a review see [119,120]).

Inhibition of DNA topoisomerases (I and II) is another well documented antitumoral strategy that leads to DNA damage, cell cycle arrest, and activation of DNA repair pathways and may result in apoptosis or other types of cell death [121,122]. Briefly, topoisomerases (Figure 5) are in charge of winding and unwinding DNA, a process required for DNA transcription and replication (for a review see [123]). Type I topoisomerases cleave only one strand of the DNA, whereas type II topoisomerases cleave both strands, generating DSBs.

Camptothecins (e.g., topotecan, SN-38, CPT-11/irinotecan) represent the better known topoisomerase I (topI) inhibitors, together with indolocarbazoles and indenoquinolines (for a review see [124,125]). Camptothecin, which is isolated from the *Camptotheca acuminata* tree, and its derivatives are non-competitive inhibitors of topI; they form a ternary complex by binding top1 and the cleaved DNA. Indolocarbazoles also prevent DNA religation, but unlike camptothecins, DNA binding can be via intercalation.

Topoisomerase II (topII) inhibitors are classified into topII poisons and topII catalytic inhibitors. TopII poisons are defined as “drugs targeting Top2 that lead to elevated levels of Top2: DNA covalent complexes”, whereas catalytic inhibitors basically block the activity of the enzyme with no involvement of DNA damage [122]. Antineoplastic application of topII inhibitors rely on the topII poison group, which includes DNA intercalators, such as anthracyclines (e.g., doxorubicin; also mentioned in Section 4.1.1.), and non-intercalators, such as podophyllotoxins (e.g., etoposide and teniposide).

#### 5.2.3. Disruption of the DNA-Damage Repair System

As mentioned above, most anticancer therapies (chemotherapeutic or radiation therapy) are based on the induction of DNA damage as the mechanism to ultimately activate cell death pathways. Nevertheless, the presence of DNA damage is not an exclusive consequence of anticancer therapies, but a condition that often takes place during complex DNA replication and translation processes, as well as during the cell lifespan. Due to the mutagenic/lethal potential of DNA damage, cells have evolved a complex net of DNA repair systems that serve to ensure DNA integrity but may also reduce the effect of many antitumoral treatments.

Depending on the type of DNA lesion, different types of repair systems are activated [126]. The main systems in mammals are the following:

- Base excision repair (BER) primarily repairs non-bulky lesions with no major distortion of the DNA double helix structure (e.g., bases with alkylation, oxidation or ring saturation damage, deaminated bases, DNA SSBs).
- Nucleotide excision repair (NER) removes damage or incorrect bases in a 24–32 base pair oligonucleotide manner and then mends the resulting gap (e.g., bulky DNA lesions with potential for blocking DNA replication).
- Transcription coupled NER (TC-NER) repairs DNA damage specifically on the RNA polymerase II-transcribed strand. Damage recognition occurs when RNA polymerase II stalls at a DNA lesion.
- Nonhomologous end joining (NHEJ) repairs DNA DSBs when they occur outside a replication fork and not in S phase.
- Homologous recombinational repair (HRR) repairs DNA DSBs when they occur at replication forks and when cells are in S phase, and contributes to the repair of DNA interstrand crosslinks.
- Mismatch repair (MMR) recognizes and repairs base-base mismatches and insertion/deletion loops that arise during DNA replication (e.g., certain damage caused by ROS and alkylating agents).

The inhibition of DNA repair systems, especially with DNA damage-inducing drug administration, seems to be a promising strategy against cancer. Until now, there are three targeted controlling/repairing systems (Figure 5): (1) cell cycle checkpoint proteins (e.g., cyclin-dependent kinase 1 and 2 (CDK1 and CDK2)), (2) PARP-1, or (3) O-6-methylguanine-DNA methyltransferase (MGMT).

Ashwell and Zabludoff [127] summarized the current knowledge regarding CDK1 and CDK2 inhibitors, such as UCN-01, XL844, and AZD7762. As described in Section 3.2.3, the activation of ATM and ATR functions as a sensor of DSBs and SSBs. CDK1 and CDK2 are ATM and ATR-downstream effectors and play a critical role in the cell cycle by promoting cell cycle arrest in order to gain time for DNA repair and cell survival. Therefore, the inability to activate CDK1 and/or CDK2 upon DNA damage provokes the activation of apoptotic pathways as the natural alternative for the cell to ensure the elimination of damaged cells.

PARP-1, which is discussed in Section 3.2.3, is the most abundant form of the PARP family and located in the nucleus, where it works as a sensor for SSBs [128]. Activation requires binding to DNA strand breaks, which is required to recruit proteins that form the BER complex [129].

The development of PARP-1 inhibitors was based on the “single strand DNA break-inducing” capacity of many of the previously described antineoplastic drugs (e.g., topI inhibitors) and can be considered the first (though still *young*) chemopotentiating agents [130]. Drew and Plummer [129] provide an updated review of PARP-1 inhibitors from different pharmaceutical companies that are already in clinical trials, including AG014699, KU59436 (AZD2281), and ABT-888.

MGMT is an essential enzyme for repairing *O*^6^-alkylguanine-based DNA lesions in normal tissues and, therefore, a key element in resistance against *O*^6^-alkylating agents, such as temozolomide or dacarbazine. MGMT is considered a *suicide enzyme* because of its mechanism of action; it transfers the DNA-damaging alkyl group to a cysteine in its own active center, which restores the guanine base on the DNA but inactivates the enzyme (for a review see [131]). Inhibition of MGMT activity represents a strategy for enhancing the effect of several antineoplastic alkylating agents and, therefore, a suitable goal to pursue. Efforts to design MGMT inhibitors have focused on the development of guanine analogues aimed at irreversibly inactivating MGMT. The first guanine analogue was *O*^6^-methylguanine [132,133]. Nevertheless, the excessive concentrations required, as well as other problems, led to the design of other alternatives, such as *O*^6^-benzylguanine [134]. *O*^6^-benzylguanine established a platform for developing other inhibitors based on molecular changes, including the addition of methyl- or bromo- groups [131] or conjugation with glucose [135]. PaTrin-2 (*O*^6^-(4-bromo-thenyl)guanine (4BTG, Patrin™, Lomeguatrib) is perhaps the most advanced MGMT inhibitor to date [136], though there is still a long way to go on this field.

#### 5.2.4. Telomere/Telomerase Targeted Anticancer Drugs

Telomeres are essential to the maintenance of chromosomal integrity and are located at the ends of chromosomes. Human telomeres consist of 5′-TTAGGG-3′ sequence tandem repeats that shorten each round of cell division due to the inability of DNA polymerase to fully replicate the end of chromosomes [137]. In most somatic cells, continuous telomere shortening triggers replicative senescence and, ultimately, cell death (for a review see [138]). Nevertheless, mammalian germline and early embryonic cells express an RNA-dependent DNA polymerase complex (telomerase) capable of adding multiple copies of the 5′-TTAGGG-3′ motif to the end of the telomere [137]. Moreover, telomerase has been measured as being over-expressed in roughly 85% of human cancers [139], which partially explains their characteristic immortal phenotype. Based on these data, telomerase inhibition represents a possible strategy for inducing cancer cell death via the activation of senescence by compounds that a) inhibit its catalytic activity (e.g., BIBR1532), b) inhibit the expression of telomerase components, or c) alter post-translational modifications of the enzyme [140].

Telomeres are mainly formed by double-stranded DNA with a short single stranded 3′ overhang located at the end of each telomere. The overhang is extremely sensitive to degradation and, at the same time, required by the telomerase as a template for further telomere elongation. The overhang is usually protected from degradation by a protein complex known as the *telosome* or *shelterin*. The telosome/shelterin complex also controls telomere elongation by regulating the accessibility of telomerase to the overhang [140].

G-quadruplexes are guanine-based secondary DNA structures of potential interest to antitumoral compound design based on the incapability of telomerases to elongate these structures [141]. Therefore, G-quadruplex-interacting agents can also be evaluated as potential anticancer compounds. Some of these compounds, including telomestatin, have been shown to induce replicative senescence, whereas others (e.g., BRACO19, RHPS4) seem to induce the apoptotic death of tumor cells via mechanisms that may involve the activation of the DNA damage response (for a review see [140,141]).

#### 5.2.5. Disruption of the Cytoskeleton Dynamism as an Anticancer Strategy

The cytoskeleton, which is formed by microfilaments, intermediate filaments, and microtubules, is a dynamic structure essential for cell shape, intracellular vesicular/organelle trafficking, and proper cell division. Inhibitors of correct microtubule assembly/disassembly are generally named *antimitotic agents* based on the relevance of microtubules to spindle formation and, ultimately, cell division. Microtubules are basically α and β-tubulin polymers, and most antimitotic agents are classified as *tubulin-interacting agents* [124].

Two major mechanisms are known for tubulin interaction-mediated microtubule disruption: (1) inhibition of tubulin polymerization, and lack of microtubule formation, and (2) stabilization of the polymerized tubulin. Agents that act through the inhibition of tubulin polymerization are those that bind to β-tubulin at the colchicine site (e.g., podophyllotoxin, 2-methoxyestradiol, and heterocyclic ketones, including some flavonoids) or interact with the vinca domain (e.g., vinca alkaloids such as vinblastine, vincristine, and vinorelbine). Taxanes (e.g., paclitaxel and docetaxel), which are diterpenes of plant origin, represent the better known group of polymer tubulin-interacting molecules [124,142]. Epothilones (epoxides, olefins, and derivatives) and discodermolide, though less known, also stabilize the polymerized tubulin structure. Other novel anti-microtubule agents that are gaining interest are dolastatin, halichondrin B, and hemiasterlin, all of which originate from marine sponge or sea hare [142,143].

Neurotoxicity caused by classical anti-microtubule agents, such as taxanes, has supported studies investigating other non-tubulin-interacting-antimitotic compounds. Inhibitors of the aurora kinases (e.g., VX-680, AZD1151), polo-like kinase 1 (PLK1) (e.g., BI 2536), kinesin spindle protein (KSP) (e.g., ispinesib), and centromeric protein E (CENPE) (e.g., GSK-923295) are under current development (for a review see [143]).

#### 5.2.6. ER-Stress Response Altering Agents

Proteasome inhibitors are the better characterized members of this group, though recent studies suggest that ER unfolded protein response molecules, such as glucose-regulated protein 78/immunoglobulin heavy-chain binding protein (GPR78/BiP), could also be suitable targets against cancer (Figure 6).

The (26S) proteasome represents the main non-lysosomal protein degradation pathway in the cell and, consequently, a crucial mechanism for protein turnover. This multicatalytic complex is composed of two main structures: the 20S particle, the core where proteolysis occurs, and the 19S particle complex, which is in charge of ubiquitinated substrate recognition and unfolding [144]. The protein degradation capacity of the proteasome relies on three major activities: (1) chymotrypsin-like activity that cleaves after hydrophobic amino acids; (2) a trypsin-like activity that cleaves after basic amino acids, and (3) a post-glutamyl peptide hydrolyzing or caspase-like activity that cleaves after acidic amino acids [145].

Proteasome inhibitors were first designed to study the proteolytic activities of this enigmatic complex. Later, the capacity of these compounds to induce apoptotic cell death in chemotherapy-sensitive and resistant cell lines, and the fact that they preferentially attack malignant cells [146] started a new field: the use of proteasome inhibitors as antitumoral drugs (reviewed by [147,148]). From a general point of view, proteasome inhibitors can be classified into two groups: (1) peptide-based inhibitors resembling proteasome substrate proteins (e.g., bortezomib (Velcade), calfilzomid (PR-171) or MG132) and (2) natural products (e.g., lactacystin or salinosporamide A (NPI-0052)).

Different compounds seem to target distinct active sites of the 20S proteasome. MG115 and MG132 belong to the group of classic reversible inhibitors of chymotrypsin-like activity. Despite possible problems with non-specificity, lactacystin (obtained from the bacteria *Streptomyces lactacystinaeus*) has been a classical compound for irreversible 20S inhibition [147]. Other compounds, such as bortezomib, reversibly inhibit chymotrypsin-like activity and caspase-like activity (though to a minor extent) via non-covalent complex formation. On the other hand, calzomib and salinosporamide Amediated inhibition of chymotrypsin-like activity is irreversible [149].

The UPR, as indicated by its name (defined in Section 4.2.2.), is a mechanism activated in the ER upon accumulation of unfolded/misfolded proteins. GPR89/BiP, PRKR-like ER kinase (PERK), inositol-requiring kinase 1 (IRE1), and activating transcription factor 6 (ATF6) are upstream from a complex signal transduction pathway that restores ER balance by (a) increasing the expression of ER chaperones; (b) arresting mRNA translation in order to temporarily decrease activity in the ER; (c) accelerating proteasome-mediated damaged protein degradation, or (d) increasing autophagic protein degradation [150].

An enhanced UPR (especially GPR78 expression) has been observed in numerous tumors to the point of being considered a novel biomarker of tumor behavior [151]. Already in 2004, a novel compound of microbial origin was isolated from a culture broth of *Streptomyces versipellis* and was capable of suppressing the activation of the GRP78 promoter. The compound was versipelostatin (Figure 5) [152]. Current GPR78 modulators include non-selective compounds, such as genistein, as well as highly selective compounds, such as versipelostatin, novel derivatives of versipelostatin, or the bacterial AB_5_ subtilase cytosine, which cleaves GPR78 at a single amino acid [151,153]. All of the compounds have high chemotherapeutic potential that must be proven in future studies.

#### 5.2.7. Kinase Inhibiting Agents

Reversible phosphorylation processes are well known mechanisms for protein activation/deactivation and, therefore, essential in signaling pathways. Proteins in charge of transferring phosphoryl groups to target proteins are termed kinases, whereas dephosphorylation processes rely on phosphatases. Moreover, the de-regulation (e.g., sustained activation) and over-expression of different kinases is involved in several pathologies, including rheumatoid arthritis, cardiovascular disease, neurodegenerative diseases, and cancer [154]. Focusing on cancer, Bcr-Abl1 is a clear example of an aberrant fusion protein as the product of a gene formed by the fusion of the partial *abl1* and *bcr* genes. Bcr-Abl1 protein is a de-regulated, constitutively activated protein with enhanced tyrosine kinase activity and involved in pro-survival and anti-differentiation pathways. Bcr-Abl1 has the capacity to inhibit tumor suppressors (e.g., p53) and to induce DNA damage. The protein is thought to be involved in the initiation steps of chronic myeloid leukemia and represents one of the first successes of protein-targeted drug synthesis (*i.e.*, imatinib) [155]. The current view of protein kinases in cancer is composed of a long list of altered enzymes and targeted compounds [156].

Cancer-related protein kinase classification includes two main groups: (1) tyrosine kinases, which phosphorylate tyrosines of the target proteins, and (2) serine-threonine kinases, which phosphorylate either serine or threonine [156,157]:

Tyrosine kinases:
  1.1. Non-receptor tyrosine kinases:
    - Abl (v-abl Abelson murine leukemia viral oncogene homolog 1)
    - Bcr-abl1 (aberrant fusion protein)
    - Focal adhesion kinase (FAK)
    - Janus kinase (JAK)
    - Src
  1.2. Receptor tyrosine kinases:
    - Anaplastic lymphoma kinase (Alk)
    - Epidermal growth factor receptor (EGFR) family
    - Fibroblast growth factor receptor (FGFR) family
    - Platelet-derived growth factor receptor (PDGFR) family, also known as MDGF, GSM, FDGF, ODGF, MDF, OBIF, T47Dfactor, and GDGF
    - Vascular endothelial growth factor (VEGFR) family
    - Insulin growth factor receptors (IGFR)
    - c-kit (CD117)
    - Flt3 (fms-like tyrosine kinase receptor-3)
    - Met (mesenchymal/epithelial transformation factor/hepatocyte growth factor receptor)
    - Ret
Serine/threonine kinases:
  - Aurora kinases
  - CDKs
  - Chk (checkpoint kinases)
  - Extracellular signal-regulated kinase 5 (ERK5)
  - Mitogen-activated protein kinase (MAPK/MEK)
  - mTOR (mammalian target of rapamycin)
  - Nek (NIMA-related kinases)
  - Phosphoinositide-dependent kinase 1 (PDK1)
  - Phosphatidylinositol 3-kinase (PI3K)
  - Protein kinase B (PKB, also known as Akt)
  - Protein kinase C (PKC) family
  - Polo-like kinases (Polo-box domain containing kinases)
  - Raf kinases
  - Ras
  - ROCKs (Rho kinases)

**Figure 7.**
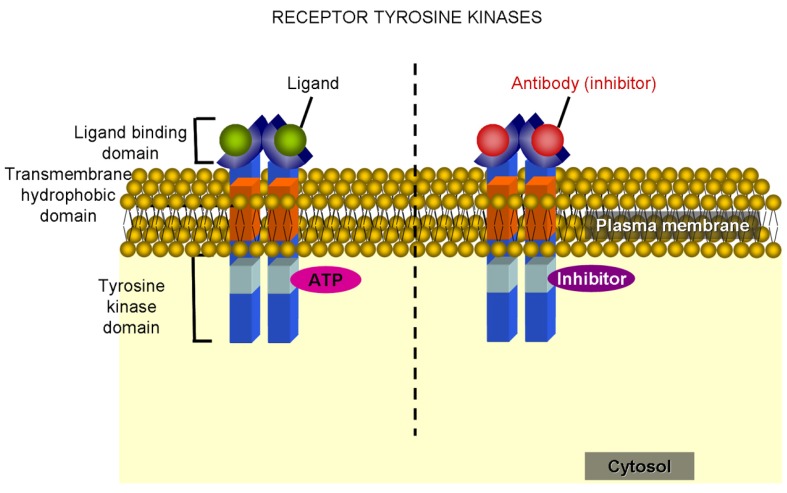
Schematic mechanism for receptor tyrosine kinase inhibition. Antibody-mediated inhibition of the ligand-binding domain and inhibition of the ATP-binding domain are main strategies for receptor tyrosine kinase inhibition.

In general, kinase inhibitors can be classified as monoclonal antibodies or targeted small molecule inhibitors. Monoclonal antibodies are theoretically suitable for targeting membrane receptor tyrosine kinases (Figure 7), though small-molecule inhibitors seem to offer more advantages, especially in regards to fewer adverse effects compared to antibodies or other common antitumor drugs [156]. The mechanism of action of small molecule inhibitors is based on affinity for either ATP-binding sites or allosteric sites. Affinity to kinase-specific allosteric sites represents the best scenario in therapy as ATP-binding sites are not exclusive to a certain kinase.

Currently available antibody-based therapies include beracizumab to block VEGFR and trastuzumab (commercially launched as Herceptin) against HER2, a member of the EGFR receptor tyrosine kinase family. Other therapies, including the EGFR blockers GA 201, pertuzumab, and zalutumumab; IGF-1R ligands AMG 479, BIIB 022, cixutumumab, figitumumab, and robatumumab; and VEGFR signaling pathway members MetMAb, SCH 900105 (against Met), alacizumab pegol, IMC 18F1, and ramucirumab, belong to different pharmaceutical companies and are undergoing clinical trials [156].

Small molecule-based strategies include a long list of compounds [156]. For simplicity, we briefly describe the rationale behind some of these antitumoral drugs:

Tyrosine kinase inhibitors are the largest group of kinase-inhibiting small molecules. Most of the compounds act, as mentioned above, by blocking the ATP-binding site of the target molecule (Figure 7) [158]. Imatinib mesylate (STI571 or Gleevec) was the first member of this group and, as mentioned above, designed to block the aberrant fusion protein Bcr-abl, which is present in nearly all patients with chronic myeloid leukemia. Moreover, imatinib has been shown to inhibit c-kit and PDGFR tyrosine kinases, which are thought to play an important role in gastrointestinal stromal tumor (GIST) formation. Unfortunately, disease relapse involving acquired resistance to imatinib has been observed in many patients and forced the development of alternative treatments, such as sunitinib [159,160]. Regarding chronic myeloid leukemia, mutations in the Bcr-abl kinase domain seem to be the most common mechanism underlying acquired resistance [161]. Dasatinib and nilotinib are also among the current alternatives for imatinib-resistant disease. Dasatinib is a non-specific Bcr-abl inhibitor capable of binding both active and inactive conformations of Bcr-abl, whereas nilotinib is an imatinib-related molecule specific to the inactive conformation of Bcr-abl [162].

Other tyrosine kinase inhibitors include EGFR inhibitors gefitinib, erlotinib (OSI-774), lapatinib (GW-572016), and canertinib (CI-1033); VEGFR inhibitors semaxinib (SU5416), vatalanib (PTK787/ZK222584), sutent (SU11248), and sorafenib (BAY 43-9006); and PDGF inhibitor leflunomide (SU101) [156,162].

Regarding serine/threonine kinases, aurora kinase (A, B, and C) inhibitors are promising antineoplastic agents. Aurora kinases are implicated in the control of chromosome assembly and segregation during mitosis and, therefore, their abnormal expression/regulation in tumor cells is closely associated with genetic instability [163]. Most inhibitors target better described members (kinase A and B), though some, such as AZD1152 (upon conversion into its active metabolite AZD1152-HQPA), present high affinity for all aurora kinases (for a review see [156,163]).

mTOR is also another target of cancer therapy due to its central position in several pro-survival pathways (e.g., PI3K/Akt/mTOR). In normal cells, mTOR is activated by either upstream positive regulators, such as growth factors, and inactivated by negative regulators, such as the phosphatase and tensin homolog (PTEN) [164]. Interestingly, mTOR and many of its regulating molecules present alterations in malignant cells, supporting the therapeutic value of mTOR inhibition. As the mammalian target of rapamycin, mTOR was identified as a rapamycin substrate [165], and rapamycin was the first inhibitor tested against different human cancer cell lines. Despite initial positive results, unfavorable pharmacokinetic properties required the synthesis of new analogues, such as temsirolimus (CCI-779), everolimus (RAD001), and ridaforolimus (AP23573 or deforolimus). All of these compounds act by forming a complex with FK506 binding protein-12 (FKBP-12), which is recognized by mTOR, resulting in the inhibition of its activity [166] (for a review see [156,164]).

#### 5.2.8. Others

This group includes other novel strategies that are still in early phases, such as inhibitors of IAPs, LMP driving compounds, and mitochondria targeted compounds (also known as *mitocans*). The mammalian IAP family is formed by a group of eight members capable of directly inhibiting caspases (already mentioned in Section 3.2.1). The release of the natural IAP inhibitor (*i.e.*, Smac/DIABLO) from the mitochondria allows caspase activation and, ultimately, apoptotic death of the cell. Baculoviral IAP repeat (BIR) domains (from 1, up to 3) are the identifying structural motifs in IAPs and in charge of mediating the interaction with caspases. In this regard, the current inhibitors of cellular IAP activity are mainly BIR2 or BIR3 antagonists (e.g., embelins, monovalent Smac mimetics, and arylsulfonamides; Figure 6) that are still in preclinical or early clinical phases (for a complete review see [167]).

Lysosomes, hydrolase-containing acidic organelle described by de Duve *et al.* [168] are gaining importance in different processes in addition to macromolecule degradation. Lysosomes contain large amounts and varieties of cathepsins (cysteine, aspartate, or serine proteases), as well as sphingolipid lipases (acidic sphingomyelinase and ceramidase). In addition, transmembrane vacuolar H^+^-ATPase, lysosome-associated membrane protein (LAMP) 1 and 2, and CD63 (LIMP-1/LAMP-3) are among the long list of proteins required for correct lysosomal function [169]. As mentioned above in Section 3.2.4, numerous cancer treatments induce direct or indirect permeabilization of the lysosomal membrane, and ultimately cell death. Groth-Pedersen *et al.* [170] presented an updated list of established and putative lysosomal cell death-inducing cancer treatments. Among those directly affecting lysosomal stability, we can highlight lysosomotrophic compounds, such as hydroxychloroquine, BAMLET, siramesine, LeuLeuOMe, and sphingosine, which allow the release of cathepsins and activate apoptotic pathways (Figure 6).

Mitochondrion has been an intriguing organelle in cancer, especially since Otto Warburg [171] postulated in 1924 that malignant tumor cells can be caused by a shift in the energy (ATP) production system. An increased non-oxidative breakdown of glucose (also called *glycolysis*) was observed in tumor cells compared to normal cells (known as the *Warburg effect*). In fact, Warburg interpreted these studies as the result of defective mitochondrial oxidative phosphorylation with the compensatory up-regulation of glycolysis [171]. Nevertheless, the increase in glycolysis-dependent energy production does not mean a lack of oxidative phosphorylation or resistance against MMP. In fact, current anticancer strategies involving mitochondria are being pursued to: a) induce direct MMP (e.g., by interfering with PTP components such as 3-PB, bisphosphonates, or CD437), b) induce indirect membrane permeabilization (e.g., through oxidative stress-driven damage), or c) inhibit the glycolytic breakdown of glucose (e.g., by blocking pyruvate dehydrogenase kinase) (for a review see [172,173]).

## 6. Conclusions

Knowledge regarding cell death/survival pathways and the characterization of the main altered pathways in cancer has allowed an exponential increase in anticancer strategies and potential antineoplastic drugs. Nevertheless, the success in obtaining a final anticancer treatment requires long-term studies and a few of the proposed compounds reaching the finishing line. Moreover, the huge variability observed in cancer represents an intrinsic challenge of the disease. With this adverse scenario in mind, movement towards an individualized characterization of the disease represents an inestimable advance, which is desirable for the future. The best chances for treatment seem to rely on a combination of the better-known broad-spectrum antitumoral drugs, together with the design of selective molecule-targeted compounds against altered regulatory molecules.

## Figures and Tables

**Table 1.** Main morphological and biochemical markers of apoptosis. Column on the left represents main morphological changes during the apoptotic process; column on the right summarizes main biochemical markers of the apoptotic cell death.

Morphological Characterization		Biochemical Characterization
Apoptosis	Rounding-up (on attached cells) Chromatine condensation Reduction nuclear volumen (pyknosis) Plasma membrane blebbing Nuclear fragmentation (karyorrhexis) Apoptotic body formation	■ Phosphatidylserine (PS) exposure ■ Activation of proapoptotic Bcl-2 family proteins (e.g. Bax, Bak, Bid) ■ Increase of reactive oxygen specie (ROS) production ■ Activation of caspases ■ Dissipation of Δ Ψ_m_
Late apoptosis	Engulfment by phagocytes (*in vivo*) Secondary necrosis (*in vitro*)	■ Mitochondrial membrane permeabilization ■ Oligonucleosomal DNA fragmentation

**Table 2.** Classification of antitumoral drugs according to their Anatomical Therapeutic Chemical (ATC) classification.

**A-Alkylating Agents**
AA-Nitrogen mustard analogues (e.g., methorethamine)
AB-Alkyl sulfonates (e.g., busulfan)
AC-Ethyelene imines or aziridines (e.g., thiotepa)
AD-Nitrosoureas (e.g., carmustine, lomustine, semuestine)
AG-Epoxides
AX-Other alkylating agents

**B-Antimetabolites**

BA-Folic acid analogues (e.g., methotrexate)
BB-Purine analogues (e.g., azathioprine, mercaptopurine)
BC-Pyrimidine analogues (e.g., 5-fluorouracil, capecitabine, cytosine arabinoside)

**C-Plant Alkaloides and Other Natural Products**

CA-Vinca alkaloids and analogues (e.g., vinblastine, vinorelbine)
CB-Podophyllotoxin derivatives (e.g., etoposide)
CC-Colchicine derivatives
CD-Taxanes (paclitaxel, docetaxel)
CX-Other plant alkaloids and natural products (e.g., camptothecin)

**D-Cytotoxic Antibiotics and Related Substances**

DA-Actinomycines (e.g., actinomycin D)
DB-Anthracyclines and related substances (e.g., doxorubicin, daunorubicin)
DC-Other cytotoxic antibiotics (e.g., mitomycin C, bleomycin)

**X-Other Antineoplastic Agents**

XA-Platinum compounds (e.g., cisplatin, carboplatin, oxaliplatin)
XB-Methylhydrazines
XC-Monoclonal antibodies (e.g., edrecolomab-antiEpCAM-, cetuximab-antiErbBl-, rituximab-antiMS4A1 (CD20 antigen), bevacizumab-antiVEGF…)
XD-Sensitizers used in photodynamic/radiation therapy
XE-Protein kinase inhibitors (e.g., imatinib, erlotinib, sunitinib, nilotinib…)
XX-Other antineoplastic agents (e.g., hydroxycarbamide, tretinoin, celecoxib…)
XY-Combinations of antineoplastic agents

**Table 3.** Classification of antitumoral drugs according to their cellular target.

**Classical Chemotherapy (without precise or known molecular target)**	**Specific Molecule-Targeted Agents**

**1. DNA damaging agents**	**1. Bcl-2 (and related molecule) targeted compounds** (e.g., AT-101, ABT-737, oblimersen sodium)
1.1. Platinum compounds (e.g., cisplatin, carboplatin, oxaliplatin)	
1.2. Alkylating agents (nitrogen mustards)	
1.3. Cytotoxic antibiotics (e.g., actinomycin D, anthracyclines, mitomycins, bleomycins)	
**2. Oxidative stress-mediated cell death inducers** (e.g., 4-HPR, PEITC)	**2. DNA damaging agents**
	2.1. Antimetabolites (e.g., methotrexate)
	2.2. DNA topoisomerase (I and II) inhibitors (e.g., camtothecins, podophyllotoxins)
	**3. Drugs to target DNA-damage repairing systems**
	3.1. CDK inhibitors (e.g., UCN-01, XL844)
	3.2. PARP-1 inhibitors (e.g., AG014699)
	3.3. MGMT inhibitors (e.g., PaTrin-2)
	**4. Telomere/Telomerase-targeted anticancer drugs** (BIBR1532, telomestatin, BRACO19)
	**5. Compounds to disrupt cytoskeleton dynamisn** (Vinca alkaloids, taxanes, epothilones)
	**6. ER-stress response altering agents Proteasome inhibitors**
	6.1. Proteasome inhibitors (e.g., bortezomib, salinosporamide A)
	6.2. Inhibitors of unfolded protein response (UPR) molecules (e.g., versipelostatin)
	**7. Kinases inhibiting agents**
	7.1. Antibody-based therapies (e.g., beracizumab, trastuzumab)
	7.2. Small molecule-based therapies (e.g., imatinib, erlotinib, AZD1152, temsirolimus)
	**8. Others**
	8.1. IAP inhibitors
	8.2. Lysosome permeabilization inducing agents
	8.3. Mitochondria-targeted compounds
